# Designing artificial ion channels with strict K^+^/Na^+^ selectivity toward next-generation electric-eel-mimetic ionic power generation

**DOI:** 10.1093/nsr/nwad260

**Published:** 2023-10-03

**Authors:** Jipeng Li, Linhan Du, Xian Kong, Jianzhong Wu, Diannan Lu, Lei Jiang, Wei Guo

**Affiliations:** State Key Laboratory of Marine Resource Utilization in South China Sea, School of Materials Science and Engineering, Hainan University, Haikou570228, China; Department of Chemical Engineering, Tsinghua University, Beijing100084, China; South China Advanced Institute for Soft Matter Science and Technology, Guangdong Provincial Key Laboratory of Functional and Intelligent Hybrid Materials and Devices, School of Emergent Soft Matter, South China University of Technology, Guangzhou510640, China; Department of Chemical and Environmental Engineering, University of California, Riverside, CA92521, USA; Department of Chemical Engineering, Tsinghua University, Beijing100084, China; Research Institute for Frontier Science, Beihang University, Beijing100191, China; Research Institute for Frontier Science, Beihang University, Beijing100191, China; Center for Quantum Physics and Intelligent Sciences, Department of Physics, Capital Normal University, Beijing100048, China

**Keywords:** artificial ion channel, bi-layer graphene, dehydrated ion transport, bio-inspired materials, energy conversion

## Abstract

A biological potassium channel is >1000 times more permeable to K^+^ than to Na^+^ and exhibits a giant permeation rate of ∼10^8^ ions/s. It is a great challenge to construct artificial potassium channels with such high selectivity and ion conduction rate. Herein, we unveil a long-overlooked structural feature that underpins the ultra-high K^+^/Na^+^ selectivity. By carrying out massive molecular dynamics simulation for ion transport through carbonyl-oxygen-modified bi-layer graphene nanopores, we find that the twisted carbonyl rings enable strict potassium selectivity with a dynamic K^+^/Na^+^ selectivity ratio of 1295 and a K^+^ conduction rate of 3.5 × 10^7^ ions/s, approaching those of the biological counterparts. Intriguingly, atomic trajectories of K^+^ permeation events suggest a dual-ion transport mode, i.e. two like-charged potassium ions are successively captured by the nanopores in the graphene bi-layer and are interconnected by sharing one or two interlayer water molecules. The dual-ion behavior allows rapid release of the exiting potassium ion via a soft knock-on mechanism, which has previously been found only in biological ion channels. As a proof-of-concept utilization of this discovery, we propose a novel way for ionic power generation by mixing KCl and NaCl solutions through the bi-layer graphene nanopores, termed potassium-permselectivity enabled osmotic power generation (PoPee-OPG). Theoretically, the biomimetic device achieves a very high power density of >1000 W/m^2^ with graphene sheets of <1% porosity. This study provides a blueprint for artificial potassium channels and thus paves the way toward next-generation electric-eel-mimetic ionic power generation.

## Teaser

Bi-layer graphene nanopores with twisted carbonyl rings possess strict K^+^ selectivity over Na^+^ as that of the biological counterparts, and open a door toward kilowatt-scale ionic power generation based on Na^+^/K^+^ selectivity.

## INTRODUCTION

**Figure 1.**
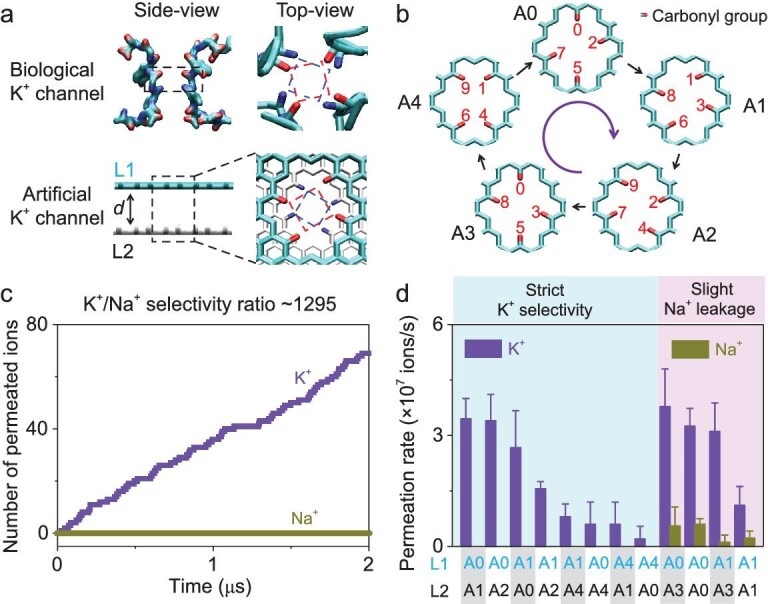
Designing an artificial ion channel with strict potassium selectivity. (a and b) Molecular structure of the selectivity filter of a KcsA potassium channel. Adjacent carbonyl rings exhibit a 27^o^ rotation. Inspired by this structural feature, an artificial potassium channel is built based on a bi-layer graphene nanopore. Four-site carbonyl modification is carried out on the pore edge in each layer and a clockwise integral twist of the functionalized positions is adopted to the nanopore in the next layer. Following this strategy, five types of single-layer nanopore elements are obtained (termed A0 to A4). (c) Ion permeation test shows strict K^+^ selectivity over Na^+^. The tested pore configuration is A0A1. (d) Among the 12 tested combinations of bi-layer nanopores, 8 types of hetero-bi-layer nanopores (A*m*A*n, m*≠*n*) show strict K^+^ selectivity. Slight Na^+^ leakage is found in the remaining four types of homo- or hetero-bi-layer nanopores.

Selective ion transport through protein channels is fundamental to cellular signal transduction [1–4]. The gated process helps to generate action potentials and other electrical signals that regulate the biological activities in living cells. For decades, selective permeation of larger K^+^ (ionic radius of 1.3 Å) over smaller Na^+^ (1.0 Å) [5,6] through potassium channels has attracted broad research interest from both biological and materials communities [7,8]. The crystal structure of the KcsA potassium channel, for example, reveals that the extraordinary K^+^/Na^+^ selectivity ratio (SR) of >1000-fold originates from a four-fold symmetric tubular selectivity filter with a length of 12 Å [9–11]. Four periodic layers of carbonyl rings with oxygen atoms pointing to the axis of the tube function as the ion binding sites [12–15]. It is worth noting that the two adjacent carbonyl rings are not strictly overlapped (Fig. 1a). Instead, there is a nearly 27^o^ rotation due to the dihedral angles of amino acids [16,17]. Although this structural feature in biological ion channels is vital for the strict K^+^ selectivity, the rotary carbonyl rings have never been utilized in constructing artificial potassium channels.

Existing artificial potassium channels to mimic the potassium-ion-specific transport behavior of biological channels are constructed using chemically modified nanoporous materials or engineered molecular assemblies residing in lipid membranes [18–20]. A variety of nanomaterials and molecular structures, such as crown ethers, cucurbituril and foldamers, have been explored for assembling artificial potassium channels [21–26]. Typically, their K^+^/Na^+^ selectivity (SR < 40-fold) falls far behind the biological counterparts. The low selectivity is often attributed to the straight tubular configuration of ion channels commonly adopted in man-made materials, through which the leak of non-specific Na^+^ becomes unavoidable [27–30]. However, a precise design of the rotary ion binding sites has never been attempted before.

The present work demonstrates a computational design of an artificial ion channels with strict potassium selectivity. As illustrated in Fig. 1a and b, our design is based on a bi-layer graphene nanopore functionalized with twisted carbonyl on the pore edges. The artificial ion channel inhibits the transport of unwanted Na^+^, even in mixed ionic solutions. The dynamic K^+^/Na^+^ SR, estimated by using the transition-state theory, is nearly 1295-fold and the K^+^ conduction rate reaches 3.5 × 10^7^ ions/s, approaching the level of biological potassium channels. The atomic trajectories of K^+^ permeation events unveil a dual-ion transport mechanism, showing that the release of a K^+^ from the exit of the bi-layer nanopore is achieved by a soft knock-on from another K^+^ at the entrance. The synergetic behavior is facilitated by hydrate water molecules between the graphene layers that establish a kinetic pathway between two confined potassium ions under meta-stable conditions. The potassium-permselective ion channel enables a novel way of osmotic power generation by mixing KCl and NaCl solutions. For a bi-layer graphene nanopore membrane with a porosity of <1%, the theoretical power density can be >1000 W/m^2^. Overall, our results elucidate how the bi-layer nanopore structure with twisted carbonyl functionalization enables strict potassium selectivity and leads to high-performance ionic power generation.

## RESULTS AND DISCUSSION

### Carbonyl modified bi-layer graphene nanopore

Inspired by the rotary distribution of carbonyl rings in the selectivity filter of biological KcsA channels [16], we establish a bi-layer graphene nanopore model with twisted carbonyl functionalization as an artificial potassium channel (Fig. 1a). The carbonyl ring structure was generated by first removing 16 carbon atoms from a graphene lattice, yielding a 9.8 × 9.9 Å^2^ sub-nanometer pore (Supplementary Fig. S1A), followed by four-site carbonyl functionalization to the pore edges. Each graphene layer mimics the potassium ion binding sites in the biological selectivity filter.

Figure 1b shows, for example, the four carbon atoms at positions 0, 2, 5 and 7 that were modified with the carbonyl group in order to yield symmetric potassium ion binding sites in each graphene layer. The configurations with two or more adjacent carbonyl groups were dismissed because of symmetry and unfavorable ion binding energy. Five types of single-layer nanopore elements (termed A0 to A4, respectively, Fig. 1b) can be obtained by taking a clockwise integral twist of the functionalized positions from the initial configuration (A0).

After carbonyl functionalization, the effective cross-sectional area reduces to ∼32 Å^2^ (Supplementary Fig. S1A), close to that of the selectivity filter of biological KcsA channels [31]. The identical strategy was used to generate nanopores in two parallel graphene layers (termed L1 and L2) with the interlayer distance set to 3.35 Å in accordance with the pristine graphite.

### Strict potassium ion permeation and sodium-ion inhibition

We carried out all-atom molecular dynamics (MD) simulations for the transport of K^+^ and Na^+^ through the bi-layer graphene pores with different pore configurations and solution conditions. In all cases, the ion transport was driven by an electric field perpendicular to the graphene surface with a strength of 10 mV/nm. The simulation results reveal that, when the nanopore takes the configuration of A0 in L1 and A1 in L2 (shorted as A0A1), K^+^ can permeate at a rate of ∼3.5 × 10^7^ ions/s (Fig. 1c), approaching that of biological potassium channels (∼10^8^ ions/s). In sharp contrast, not even a single Na^+^ was permeated through the nanopore in the entire simulation time, showing strict potassium selectivity for single electrolyte solutions. In mixed solutions containing both K^+^ and Na^+^, the bi-layer nanopore also exhibits perfect K^+^ permeation and Na^+^ inhibition (Supplementary Fig. S2).

The extremely low Na^+^ permeability prohibits the direct estimation of the ion permeation rate through MD simulation with a statistical significance. As gleaned from the detailed examination of the K^+^ permeation events, the rate-determining step of the translocation process is the ion hopping from its meta-stable position at the first layer of the graphene to that at the second layer (see the next section). We estimate the permeation rates of both K^+^ and Na^+^ based on the single-ion potential of mean force (PMF) and the transition-state theory (see Methods and Supplementary Text S3). The K^+^/Na^+^ selectivity is related to the ratio of their permeation rates, which reaches 1295-fold, even surpassing that of the biological potassium channel (∼1000-fold).

The five types of single-layer nanopore elements lead to 25 combinations of bi-layer nanopore configurations (Supplementary Table S1). Excluding the duplicated combinations due to the symmetry, we investigate the permeability of K^+^ and Na^+^ through the remaining 13 bi-layer nanopores. The MD simulations were performed under identical conditions to those used for Fig. 1c. As summarized in Fig. 1d, eight types of hetero-bi-layer nanopores (A*m*A*n, m*≠*n*) show strict K^+^ permeation and Na^+^ inhibition, in spite of differing K^+^ permeability. The other four types of homo- or hetero-bi-layer nanopores show slight Na^+^ leakage. Even in these cases, their K^+^ permeability is still 5–28 times higher than that of Na^+^. More significant Na^+^ leakage is found in single-layer nanopores (Supplementary Text S5). The static K^+^/Na^+^ SR reaches merely ∼3-fold, in agreement with previous studies [32–35]. For the bi-layer nanopore with configuration A4A4, neither K^+^ or Na^+^ permeation is observed over the entire duration of the MD simulation.

Based on these results, we conclude that the hetero-bi-layer nanopore structures are responsible for the strict potassium selectivity. First, the bi-layer structure prevents the transmission of interfering Na^+^ as observed for the single-layer nanopores. Second, the selectivity and permeation rate can be optimized by using a precise design of the twisted carbonyl groups at the nanopore terminals. In addition, even with flexible carbonyl groups on the pore edge, strict K^+^/Na^+^ selectivity can be equally held (Supplementary Text S6).

### Dual-ion transport mechanism

**Figure 2.**
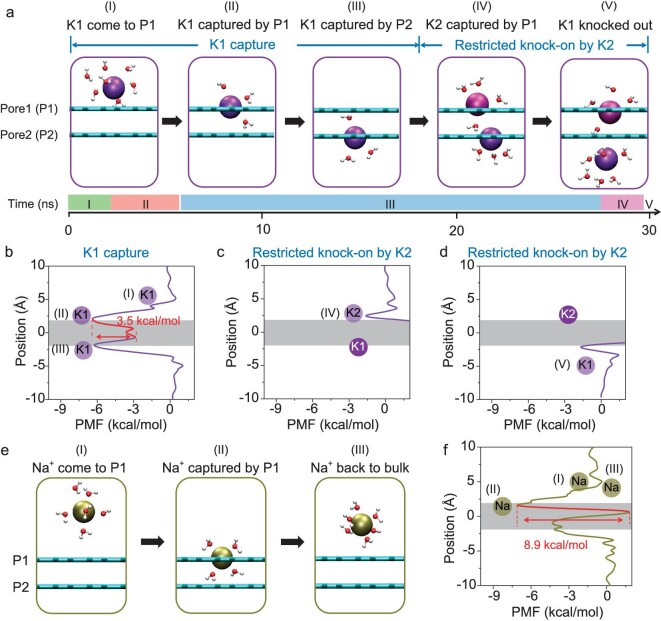
Dual-ion transport mechanism. (a) Snapshots of the representative stages on the timeline for one chosen K^+^ permeation, including the single-ion stages (I, II, III) and the dual-ion stages (IV, V). (b) Potential of mean force (PMF) of a single potassium ion (termed K1) permeating through the bi-layer nanopore in the Stages I to III mentioned in (a). (c) PMF of the second potassium ion (termed K2) invading the bi-layer nanopore with a K1 already captured by P2. (d) PMF of K1 leaving P2. (e and f) Snapshots and PMF of the representative stages for the blockade of Na^+^ permeation. In (b–d and f), shadow regions indicate the positions of the bi-layer nanopore.

The simulation results indicate that only cations can pass through the bi-layer nanopores. Anion transport is prohibited due to the small pore size and the negative charges carried by the oxygen atom of each carbonyl group. By tracing the trajectories of K^+^ permeation through the bi-layer nanopores, we find that each K^+^ permeation event involves at least two potassium ions, exhibiting a dual-ion transport mechanism. Figure 2a depicts the representative stages of a potassium ion passing through the nanopore. Based on the number of participating potassium ions, we divide the dual-ion transport into two sub-processes, termed ‘K1 capture’ (Fig. 2a, Stages I–III) and ‘restricted knock-on by K2’ (Fig. 2a, Stages IV and V).

Far from the graphene sheets, potassium ions move freely in the bulk solution (Fig. 2a, Stage I). At this stage, there is hardly any interaction between the dissolved ions and the nanopore due to the large distance. Under an external electric field, a potassium ion (denoted as K1) gets close to the nanopore in L1 (termed as P1) and it is rapidly captured by P1 due to coulombic attraction (Fig. 2a, Stage II). The binding affinity is ∼6.0 kcal/mol according to the PMF (Fig. 2b). Ion capture by the nanopore results in K^+^ partial dehydration due to the small pore size and carbonyl substitution. The hydration number of the captured potassium ion falls from 7.3 to 3.1 (Supplementary Fig. S7A), i.e. K^+^ at P1 is hydrated by a single water molecule between the graphene layers and only two water molecules on the graphene surface.

Instead of retreating back to bulk solution, K1 moves forward to the nanopore in L2 (termed as P2, Fig. 2a, Stage III) driven by the external electric field. The energy barrier from Stages II to III is merely 3.5 kcal/mol, due to the strong interaction between K1 and the carbonyl groups, and it is also far lower than that for going back to the bulk solution (6.0 kcal/mol). Once K1 resides in P2, it cannot escape the nanopore by itself due to the high energy barrier (∼6.4 kcal/mol) between Stage III and the bulk solution (Fig. 2b).

While K1 is trapped in Stage III, the vacant P1 is capable of capturing another K^+^ (denoted as K2), leading to a different energy state for K1 in P2 (Fig. 2a, Stage IV). Although the incoming K2 encounters strong coulombic repulsion from K1, the capture of K2 by P1 is thermodynamically favorable. A negative free energy change of about −1.6 kcal/mol is found in the dual-ion PMF (Fig. 2c), meaning that, with K1 at P2, the unoccupied P1 is still attractive to potassium ions. Conceivably, the intake of K2 is much slower in comparison with that for K1 when the nanopore is free of potassium ions. The simulation results show that the occupation of P1 by K2 takes almost >70% of the total time for an individual ion permeation event (see the timeline in Fig. 2a).

With two potassium ions occupied in the nanopores (Stage IV), the strong coulombic repulsion makes the dual-ion system highly unstable. The center-to-center distance between the two carbonyl-stabilized potassium ions is merely 3.9 Å, rendering a direct electrostatic energy of ∼85 kcal/mol. The strong repulsion lifts the free energy valley for K1 from −6.4 to −1.7 kcal/mol, thereby facilitating the rapid transition of K1 from Stages IV to V (Fig. 2b and d). Via this dual-ion mechanism, K1 is knocked out of P2 within ∼2.2 ns after the capture of K2 by P1 (Fig. 2a, Stage V). The process is analogous to the soft knock-on mechanism in biological ion channels [10].

In contrast to K^+^, Na^+^ stops after being captured by P1, without going forward to the next layer (Fig. 2e, Stages I and II) due to a much larger free energy barrier of 8.9 kcal/mol (Fig. 2f). Instead, the captured Na^+^ is more likely to retreat back to bulk solution (Fig. 2e, Stage III) because the retreating free energy barrier is 2 kcal/mol lower than that needed to jump from P1 to P2. The inability of Na^+^ permeation can also be attributed to its solvation environment. The hydration number of the captured Na^+^ is still >3.0, suggesting that the four carbonyl oxygens on P1 do not properly coordinate with Na^+^ (Supplementary Fig. S7B) due to its small size.

### Role of K–*n*H_2_O–K triplets

The water molecules confined between the graphene layers play an important role in the transport of potassium ions through the bi-layer nanopore. By tracing several hundreds of dual-ion transport events, we find that the dual-ion configuration is mediated by one or two hydrate water molecules between the two potassium ions (see e.g. Fig. 2a, Stage IV). The dual-ion PMF reveals that K1 and K2 are subject to a mutually attractive potential (Fig. 2c and d) due to the interlayer water molecules.

**Figure 3.**
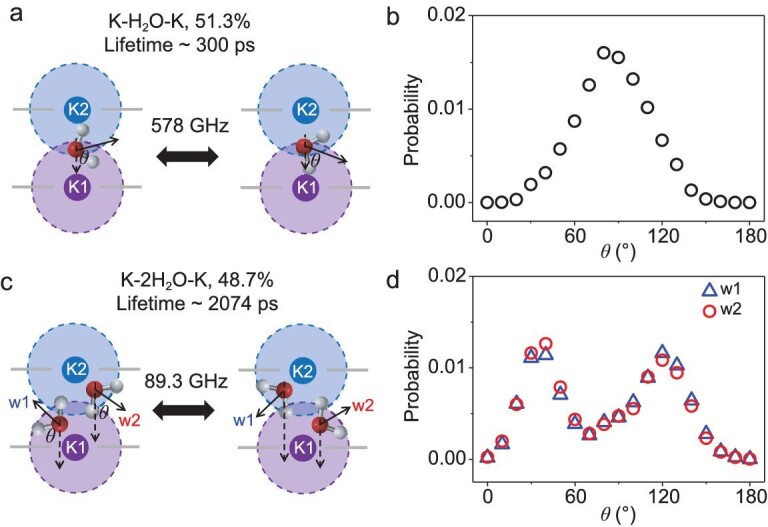
K–*n*H_2_O–K structure. (a) Schematic illustration of a K–H_2_O–K triplet. It exists in 51.3% of the K^+^ permeation events with an average lifetime of ∼300 ps. The intermediate water molecule switches between two possible orientations with a frequency of 578 GHz. (b) Probability distributions of water dipole orientation in K–H_2_O–K triplets. (c) Schematic illustration of a K–2H_2_O–K triplet. It exists in the remaining 48.7% of the K^+^ permeation events with an average lifetime of ∼2074 ps. The two intermediate water molecules switch between two possible coordination states with a frequency of 89.3 GHz. (d) Probability distributions of water dipole orientation in K–2H_2_O–K triplets.

We systematically investigate the structural and kinetic behavior of the ion–water–ion triplets based on the number of hydrate water molecules between the two potassium ions, termed as K–H_2_O–K and K–2H_2_O–K, separately. As shown in Fig. 3a, K–H_2_O–K triplets are involved in 51.3% of all dual-ion transport events. Their mean lifetime is merely 300 ps, indicating a fast de-structuring process. The atomic trajectories show that a single water molecule exhibits a rapid switching between the two ions at a frequency of 578 GHz. Statistical results on the dipole direction show a normal distribution centered at ∼90° (Fig. 3b), suggesting that the water molecule has no preferable affiliation with either K1 or K2. The solvation frustration [36], i.e. a single water molecule cannot solvate both potassium ions concurrently, makes the K–H_2_O–K triplet energetically unfavorable. In other words, the K–H_2_O–K triplets are prone to disintegration by knocking out K1 from P2.

Figure 3c shows representative atomic configurations for K–2H_2_O–K triplets that are involved in the remaining 48.7% of the potassium permeation events. They hold a much longer mean lifetime of ∼2074 ps in comparison with K–H_2_O–K. In addition, the mean electric energy of a K–2H_2_O–K triplets is 9.2 kcal/mol lower than that of K–H_2_O–K (Supplementary Fig. S8). These results suggest that K–2H_2_O–K is more stable than K–H_2_O–K. The atomic trajectories show that the two interlayer water molecules (termed w1 and w2) bind with the two potassium ions in a cooperative manner. Their dipole directions show a very close double peak distribution centered at ∼40° and ∼120° (Fig. 3d), suggesting that each water molecule binds with only one K^+^ at a time, i.e. when w1 is affiliated with K1, w2 is affiliated with K2, and vice versa. The K–2H_2_O–K triplet is only a thermodynamically meta-stable state because w1 and w2 switch between the two potassium ions with a frequency of ∼89.3 GHz. The two-water switching frequency is much lower than that of the single-water oscillation in the case of K–H_2_O–K (578 GHz).

### Influence of interlayer spacing

**Figure 4.**
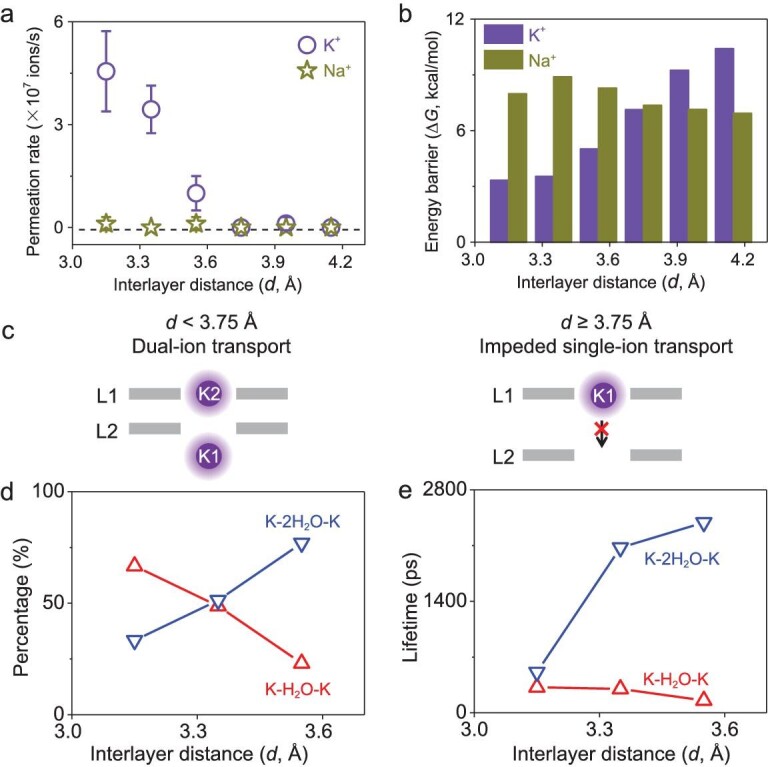
Influence of interlayer distance. (a and b) Ion permeation rate and corresponding energy barrier under different interlayer distances. (c) Schematic illustration of the transport: under a smaller interlayer distance of <3.75 Å, it follows the dual-ion transport mode, but under a larger interlayer distance, the ion cannot jump to the nanopore in the second layer, so the single-ion transport mode is impeded. (d and e) Fraction and lifetime of the K–H_2_O–K and K–2H_2_O–K triplets under varied interlayer distances.

The simulation results indicate that the interlayer distance has major effects on the permeability of K^+^. However, it has little influence on the transport of Na^+^ because it is nearly impermeable regardless of the separation between the graphene layers. As shown in Fig. 4a, the K^+^ permeation rate drops remarkably as the interlayer distance (*d*) increases. A cut-off distance can be identified at *d* ≈ 3.75 Å, above which no K^+^ permeation is observed. In contrast, no Na^+^ permeation is found in the whole test range from 3.15 to 4.15 Å.

The PMF calculated from MD simulation suggests that K^+^ permeation encounters a much higher energy barrier as the interlayer distance increases (Fig. 4b). In contrast to potassium transport, the energy barriers for Na^+^ are much larger and less sensitive to the interlayer distance. At an interlayer distance of 3.75 Å, K^+^ and Na^+^ encounter a similar energy barrier of ∼6.9 kcal/mol. Under such circumstances, both K^+^ and Na^+^ cannot get through the nanopore. The atomic trajectories show that, at large interlayer distances, K^+^ is unable to reach P2, i.e. the single-ion transport mode is impeded in the bi-layer nanopores (Fig. 4c). In other words, successful ion permeation through an associative nanopore must follow the dual-ion mechanism.

To further understand how the interlayer distance affects K^+^ permeability, we also analyse the percentages and lifetimes of different K–*n*H_2_O–K complexes. With a small interlayer distance of 3.15 Å, we find that ≤70% of the K^+^ permeations are mediated by a K–H_2_O–K triplet, while the remaining are governed by a K–2H_2_O–K triplet (Fig. 4d). Interestingly, the percentage of K–H_2_O–K dramatically declines with increasing interlayer distance. This trend is explained by the fact that more water molecules can be accommodated between the two potassium ions in the bi-layer nanopore when the interlayer distance grows.

As discussed above, the K–H_2_O–K-mediated dual-ion transport is a fast process and the triplet complex has a short lifetime of several hundred picoseconds (Fig. 4e). By contrast, the K–2H_2_O–K-mediated transport is much slower and the complex has a much longer lifetime of >2000 ps. The difference is even more dramatic when the interlayer distance increases (Fig. 4e). Based on the results of this and the above sections, we conclude that ion permeability through the bi-layer nanopores is determined by the relative content and kinetic properties of the K–*n*H_2_O–K triplets.

### A criterion for K^+^/Na^+^ selectivity

As suggested by the PMF profile (Fig. 2b) and trajectory analysis of permeation events (Fig. 2a), the free energy barrier (Δ*G*) in the middle of the bi-layer nanopore is the rate-determining step for ion permeation. To further understand the origin of K^+^ selectivity, we decompose the energy barrier into different contributions:


(1) \begin{eqnarray*}
{\mathrm{\Delta }}G = {\mathrm{\Delta }}{G}_{i{\mathrm{on}} - {\mathrm{water}}} + {\mathrm{\Delta }}{G}_{{\mathrm{carbonyl}}} \!+\! {\mathrm{\Delta }}{G}_{{\mathrm{ion}} - {\mathrm{membrane}}}.
\end{eqnarray*}

In Equation (1), Δ*G*_ion__–__water_ accounts for the free energy change due to water stripping; it is approximately proportional to the change in the hydration number (Δ*N*_water_). Δ*G*_carbonyl_ represents the free energy change related to carbonyl substitution, i.e. the replacement of water by the carbonyl group in the solvation shell of the ion. Δ*G*_carbonyl_ is proportional to the change in the number of carbonyl groups in the ion solvation shell, Δ*N*_carbonyl_. Δ*G*_ion__–__membrane_ represents the contribution by bi-layer nanopores other than the carbonyl groups and it can be treated as a constant for both K^+^ and Na^+^.

We regress Δ*G* for each ion permeating through each bi-layer nanopore of A*m*A*n* (*m* = 0 or 1; *n* = 0–3, Supplementary Fig. S9) as a function of Δ*N*_water_ and Δ*N*_carbonyl_, which can be sampled from simulation trajectory. The regression gives the contribution by the carbonyl substitution as:


(2) \begin{eqnarray*}
{\mathrm{\Delta }}{G}_{{\mathrm{carbonyl}}} \!( {{{\mathrm{K}}}^ + } ) = - 2.55 \times {\mathrm{\Delta }}{N}_{{\mathrm{carbonyl}}} < 0,
\end{eqnarray*}

(3) \begin{eqnarray*}
\Delta {G}_{{\mathrm{carbonyl}}} ( {{\mathrm{N}}{{\mathrm{a}}}^ + }) = + 4.92 \times {\mathrm{\Delta }}{N}_{{\mathrm{carbonyl}}} > 0.
\end{eqnarray*}

The negative Δ*G*_carbonyl_ (K^+^) indicates that the interaction between the nanopore and K^+^ assists ion hopping from the first to the second layer. It compromises the positive Δ*G*_ion__–__water_ due to the stripping of water molecules, leading to a relatively low energy barrier. In contrast, the positive Δ*G*_carbonyl_(Na^+^) is disadvantageous to ion hopping. When it cooperates with Δ*G*_ion__–__water_, the total energy barrier is sufficiently large to stop Na^+^ hopping to the next layer.

### Ionic power generation

**Figure 5.**
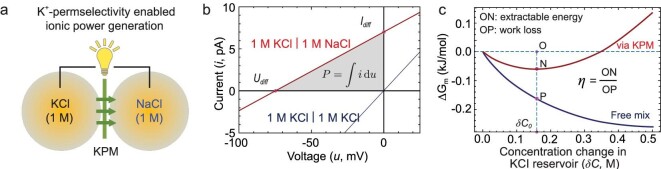
Ionic power generation. (a) Schematic illustration of ionic power generation by mixing 1 M KCl and 1 M NaCl solutions through a K^+^-permselective membrane (KPM). Only potassium ions can penetrate KPM, thus the net diffusive ionic current is from the KCl reservoir to the NaCl reservoir. (b) Current–voltage response with 1 M KCl and 1 M NaCl placed on the two sides (red). Evident non-zero intercepts are found on the two axes, suggesting net diffusive ionic current (*I*_diff_) and reversal potential (*U*_diff_). The gray area represents the net electrical power generated by the mixing process. As a control test, when equivalent molars of KCl solutions were placed on the two sides, the current–voltage response almost goes through the origin (dark blue). (c) Free energy change (Δ*G*_m_) with respect to the transferred amount of K^+^ via a KPM (red) or via a non-selective membrane (termed free mix, dark blue). In the former case, spontaneous mix can only occur before reaching a critical δC_0_ of 0.16 M. During this process, ON represents the extractable work with a KPM, while OP represents the work loss during free mix. The energy conversion efficiency is therefore defined as *η =* ON/OP.

We further explore the possibility of ionic power generation by mixing KCl and NaCl solutions through the potassium-permselective bi-layer nanopores (Fig. 5a). As shown in Fig. 5b, with 1 M KCl and NaCl solutions placed on the two sides of a bi-layer nanopore membrane, respectively, non-zero intercepts are discovered on both current and voltage axes, indicating the presence of a net diffusive ionic current (*I*_diff_) and reversal potential (*U*_diff_). In contrast, once 1 M KCl solutions were placed on both sides, the current–voltage response almost goes through the origin (Fig. 5b and Supplementary Fig. S10). Interestingly, the direction of *I*_diff_ is from the KCl reservoir to the NaCl reservoir because only K^+^ can go across the nanopore. The magnitude of *U*_diff_ reaches ∼73.1 mV, which can be quantitatively predicted by using a modified Goldman–Hodgkin–Katz theory (Supplementary Text S11). This evidence suggests that the permselective transport of K^+^ converts the Gibbs free energy loss during mixing into the ionic power. The simulation results indicate that the generated power from a single bi-layer nanopore is ∼0.13 pW by mixing 1 M KCl and NaCl solutions. Considering that the geometric pore size is <1 nm, an ultra-high pore density of 10^16^ pores/m^2^, for example, can be readily obtained, i.e. only one sub-nanometer-wide pore exists in a graphene lattice of size 10 nm × 10 nm. By exploiting parallelization [37], the theoretical power density would reach up to >1000 W/m^2^ under a low porosity of <1%.

The potassium-permselectivity enabled osmotic power generation (PoPee-OPG) better mimics the way of energy conversion by the electrocyte cells of electric eels [38] and offers major advantages over existing salinity gradient power generation (SGPG) [39,40]. First, PoPee-OPG replaces charge selectivity in SGPG with potassium ion selectivity. K^+^ goes through its exclusive permselective ionic channels. Second, PoPee-OPG eliminates the need for a low-concentration ionic solution that is indispensable in SGPG. It is worth noting that there is actually no apparent low-concentration part in the body of electric eels due to the balance of osmotic pressure across cell membranes. This improvement is equivalent to largely reducing the internal resistance of an artificial ionic power source and thus enables high output power [41].

### Energy conversion efficiency

We establish a thermodynamic process to elucidate the energy conversion mechanism and estimate the efficiency [42–44]. As schematically shown in Fig. 5a, two reservoirs of volume 1 L containing KCl (left) and NaCl (right) solutions were separated by using a bi-layer nanopore membrane. Initially, their ionic concentrations were both 1 M. If the membrane is non-selective (free mixing, Fig. 5c), the transfer of δ*C* mol of K^+^ to the right reservoir is unavoidably accompanied by a transfer of an equal amount of Na^+^ in the reverse direction. This leaves the left reservoir a mixture of (1-δ*C*) M KCl and δ*C* M NaCl, and the right reservoir a mixture of (1-δ*C*) M NaCl and δ*C* M KCl. The corresponding free energy change (Δ*G*_m_) is a decaying function with respect to δ*C* (Fig. 5c), reflecting the spontaneity of free mixing.

With a K^+^-permselective membrane (KPM) in between, Na^+^ cannot permeate. The concentration of NaCl in the left and right reservoirs remains at 0 and 1 M, respectively. With a transfer of δ*C* mol K^+^ from the left to the right reservoir, the salt content on the left is (1-δ*C*) M KCl and that on the right is a mixture of 1 M NaCl and δ*C* M KCl. The corresponding Δ*G*_m_ shows a nonmonotonic dependence on δ*C* (Fig. 5c), yet is always above the case of free mixing. Before reaching the minimum of this curve at a critical δC_0_ of ∼0.16 M, the diffusion of K^+^ is spontaneous, as the free energy change keeps declining.

For any given δ*C* ≤ δC_0_ in Fig. 5c, ON represents the extractable energy with a KPM. At the terminus of the spontaneous process, the maximum extractable energy approaches 0.06 kJ/mol. For comparison, the work loss of transferring equal amounts of K^+^ via a free mix process should be 0.16 kJ/mol (OP). Then, the energy conversion efficiency can be defined as *η =* ON*/*OP and corresponds to 37.5% at δ*C* = 0.16 M.

The efficiency of PoPee-OPG can be comparable to some of the traditional fossil and renewable energy sources (Supplementary Table S2). Compared with the ion-exchange-membrane-based SGPG, PoPee-OPG is more suitable for working in high-concentration environments. Although the efficiency of PoPee-OPG may not be as high as that of SGPG (Supplementary Table S2), the total power density of >1000 W/m^2^ reported in this work is very impressive among existing ionic energy conversion systems [40].

## CONCLUSION

In summary, we propose a computational design of an artificial potassium channel with strict potassium permselectivity. We find that twisted carbonyl modification of a bi-layer graphene nanopore can achieve an extraordinarily high K^+^/Na^+^ SR of 1295 and K^+^ permeation rate of 3.5 × 10^7^ ions/s, approaching the level of biological potassium channels. An interlayer hydrate-water-molecule-mediated dual-ion transport mechanism is responsible for the strict potassium selectivity. More importantly, as a proof-of-concept application of the potassium-permselective ionic channels, we propose a new way for ionic power generation without the need for an ionic concentration difference on the two sides of the membrane, termed as potassium-permselectivity enabled osmotic power generation. A very high power density of >1000 W/m^2^ can be theoretically achieved merely with a low porosity of <1%. The unique pore structure proposed in this work provides valuable insights for synthesizing, for example, bi-layer covalent-organic framework membranes with precisely designed ion binding sites that could lead to the development of high-performance electric-eel-mimetic energy devices [45].

## METHODS

### MD simulations

MD simulations were performed using NAMD 2.14 with a time step of 2 fs [46]. The interaction parameters were derived from a CHARMM 27 force field with TIP3P water [47]. The parameters for carbonyl groups were derived from glutamic acid. The cut-off length was 10 Å. A particle mesh ewald method was employed to compute electrostatic interaction [48]. Periodic boundary conditions were imposed in all directions. The system was equilibrated in a NP*_x_*A*_y_*_-_*_z_*T ensemble with constant area in the *y*–*z* plane and constant pressure of 1 atm. The temperature was 300 K with a Langevin damping thermostat. After equilibration, an NVT ensemble was used for the production run.

Ion permeability was calculated using non-equilibrium MD. The simulation box was 6.6 × 2.6 × 2.7 nm^3^ (Supplementary Fig. S1B), containing 1484 water molecules, 15 K^+^ (or Na^+^) and 15 Cl^−^. The salt concentration was ∼0.5 M. An electric field of 10 mV/nm was applied along the *x*-direction. The sampling was run for 900 ns.

### PMF calculation

PMFs were constructed with adaptive-biasing forces [49]. The sampling used seven uniform windows that were 3 Å thick. The spring constant of the biasing force was 100 kcal/mol. To prevent ion bumping, a harmonic constraint of 500 kcal/mol was applied to confine ion movement in a cylinder with a radius of 5 Å. A series of radii was tested to exclude possible artificial effects (Supplementary Fig. S11). Dual-ion PMFs were constructed with one ion constrained in L2.

### Transition-state theory

Due to the very low permeability, the transition-state theory was used to estimate the permeation rate of Na^+^. Based on single-ion PMF [50], the rate-determining step is the ion hopping from L1 to L2. The permeation rate is given by:


\begin{eqnarray*}
k = {\tau }^{ - 1}{e}^{ - \beta \Delta G},
\end{eqnarray*}

where *β* is the inverse thermodynamic temperature; *τ* is the attempt frequency, estimated by using the inverse of a typical vibration period of the adsorbed ion; and Δ*G* is the free energy barrier. The vibration period was estimated as the second zero point of the velocity autocorrelation function. The selectivity was calculated as the ratio of the permeation rate of K^+^ over Na^+^.

### Ionic power generation

To investigate the power generation, two graphene sheets were placed at both ends of the simulation box, forming two reservoirs connected by the nanopore (Supplementary Fig. S12). The interlayer spacing was 3.15 Å. The dimension was 60 × 2.6 × 2.7 nm^3^, containing 12 503 water molecules, 120 K^+^, 120 Na^+^ and 240 Cl^−^. The concentrations for KCl and NaCl solution were ∼1 M. To minimize the influence of net dipole, we tacitly moved the ion back to its original reservoir, corresponding to a constant concentration difference. The simulations lasted for 120 ns.

### Free energy of the mixing process

We calculated the free energy of neutral solutions using a molecular thermodynamic model [44,51]. Electrolyte solutions were described by the primitive model. Hard-sphere repulsion, direct electrostatic interactions and electrostatic correlations were treated by using the Mansoori–Carnahan–Starling–Leland equation, mean-field approximation and the mean spherical approximation (Supplementary Text S15). The hydrated diameters of K^+^, Na^+^ and Cl^−^ were 5.6, 4.7 and 6.4 Å [6].

## Supplementary Material

nwad260_Supplemental_File
