# Supplementary material for: Designing artificial ion channels with strict K+/Na+ selectivity toward next-generation electric-eel-mimetic ionic power generation
Source: Natl Sci Rev. 2023 Oct 3;10(12):nwad260. doi: 10.1093/nsr/nwad260 (PMC10632797; doi:10.1093/nsr/nwad260)
Supplement: nwad260_Supplemental_File [file nwad260_supplemental_file.pdf]

Supplementary Data

Designing artificial ion channels with strict K^+/Na^+ selectivity toward the-next-generation electric-eel-mimetic ionic power generation

Jipeng Li[1,†], Linhan Du[2,†], Xian Kong[3,*], Jianzhong Wu[4], Diannan Lu[2], Lei Jiang[5], and Wei Guo[5,6*]

[1] State Key Laboratory of Marine Resource Utilization in South China Sea, School of Materials Science and Engineering, Hainan University, Haikou 570228, China.

[2] Department of Chemical Engineering, Tsinghua University, Beijing 100084, China

[3] South China Advanced Institute for Soft Matter Science and Technology, Guangdong Provincial Key Laboratory of Functional and Intelligent Hybrid Materials and Devices, School of Emergent Soft Matter, South China University of Technology, Guangzhou 510640, China.

[4] Department of Chemical and Environmental Engineering, University of California, Riverside, California 92521, United States.

[5] Research Institute for Frontier Science, Beihang University, Beijing 100191, China.

[6] Center for Quantum Physics and Intelligent Sciences, Department of Physics, Capital Normal University, Beijing 100048, China.

* Correspondence to: wguo@iccas.ac.cn (Wei Guo); xk@scut.edu.cn (Xian Kong)

† These authors contributed equally to this work.

This PDF file includes:

Supplementary Texts 1-15

Supplementary Figures 1-12

Supplementary Tables 1-2

References

Table of contents

Supplementary Text 1. Bi-layer graphene nanopores and simulation system.

Supplementary Text 2. Ion permeation in mixed solutions.

Supplementary Text 3. Permeation rate estimated by transition state theory.

Supplementary Text 4. Possible bi-layer nanopore configurations.

Supplementary Text 5. Ion permeation through single-layer nanopore.

Supplementary Text 6. Influence of flexible carbonyl groups on pore edge.

Supplementary Text **7**. Coordination number of K^+ and Na^+.

Supplementary Text 8. Electrostatic interaction energy of K-H_2O-K and K-2H_2O-K.

Supplementary Text 9. Energy barrier of bi-layer nanopores.

Supplementary Text 10. Ionic power generation.

Supplementary Text 11. Estimation of reversal potential by a modified Goldman-Hodgkin-Katz theory.

Supplementary Text 12. Comparison of the efficiency.

Supplementary Text 13. Effect of confining cylinder radius on PMF.

Supplementary Text 14. Simulation system for ionic power generation.

Supplementary Text 15. Free energy change of mixing solutions.

References.

Supplementary Text 1. Bi-layer graphene nanopores and simulation system.

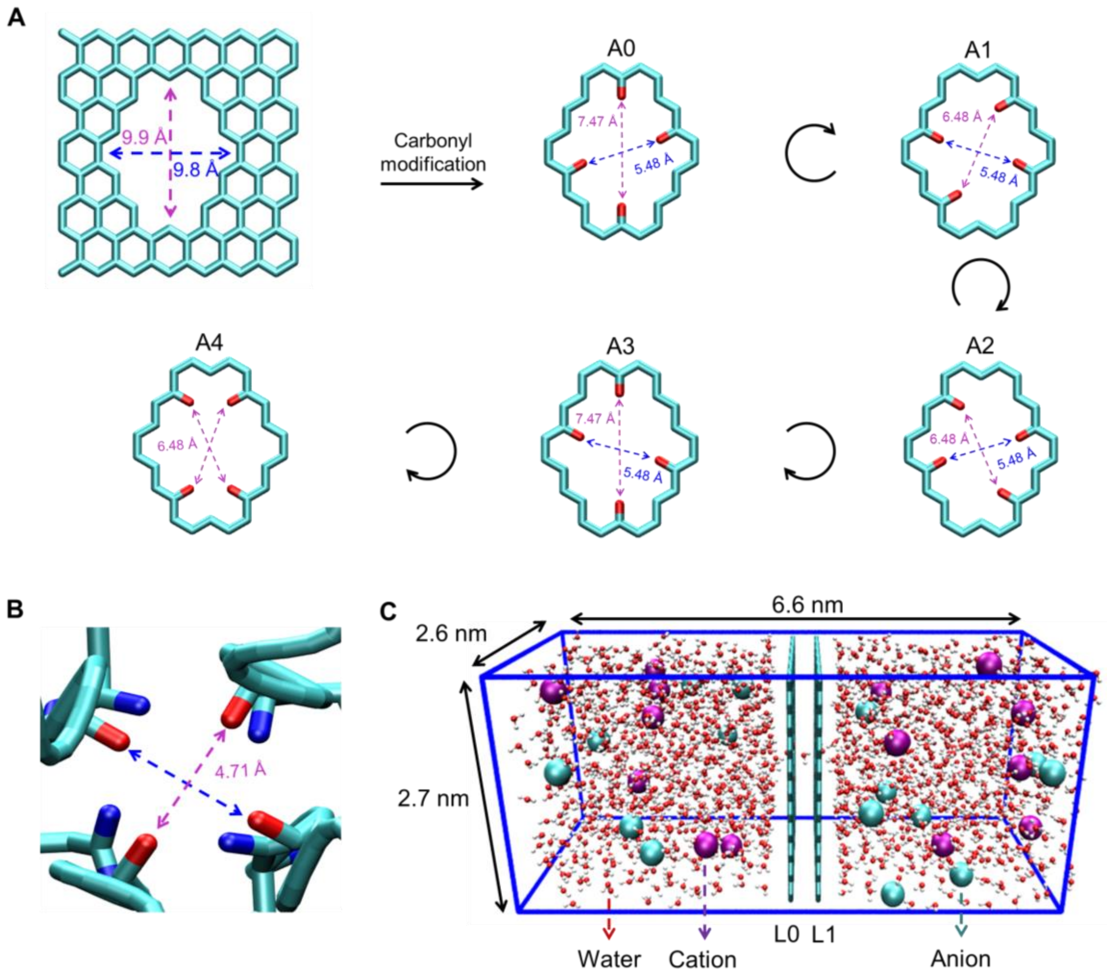

Supplementary Figure 1. Graphene nanopores and simulation setup. (A) Unmodified nanopore by removing 16 carbon atoms has a characteristic size of 9.8 × 9.9 Å^2. Symmetric 4-site carbonyl oxygen modification have only five possible choices. The resulting five types of single-layer nanopore are termed as A0, A1, A2, A3, and A4. From A0 to A4, there is a clockwise rotation in the carbonyl oxygen sites. (B) Top view of the selectivity filter of a biological potassium channel. (C) The simulation box.

Supplementary Text 2. Ion permeation in mixed solutions.

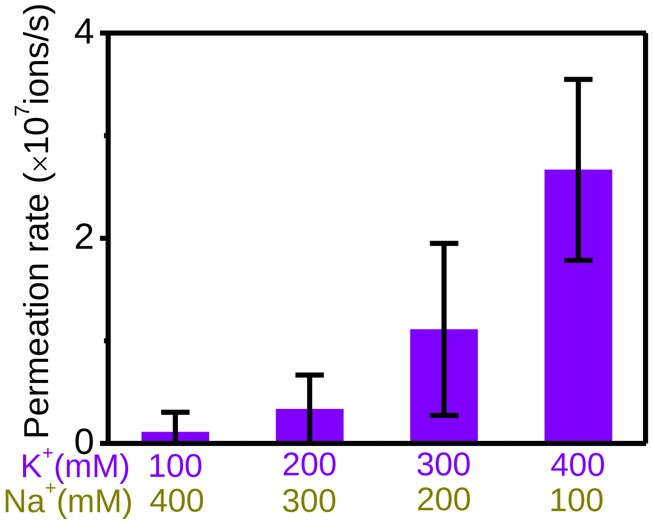

Supplementary Figure 2. Permeation of potassium and sodium ion in mixed solutions of KCl and NaCl. The total concentration was fixed at 500 mM. The concrentration of K^+ was 400, 300, 200, 100 mM. Only K^+ can permeate the bilayer nanopore with mixed solution as the feed. This means the strict K^+ selectivity of the bilayer nanopore is preserved in mixed solutions. The (almost)-linear decrease in the permeation rate of K^+ as the fraction of KCl decreases suggest that the presence of Na^+ has little interferences on the K^+ permeation.

Supplementary Text 3. Permeation rate estimated by transition state theory.

According to the transition state theory, the permeation rate can be estimated by,

$$k = \tau^{-1} e^{-\beta \Delta G}$$

where β is the inverse thermodynamic temperature, τ^{-1} the attempt frequency or the inverse of vibration period, ΔG the free energy barrier. To sample τ, we conducted simulations with the ion positioned at the first valley initially (Supplementary Fig. 3A). The vibration period τ was estimated as the second zero point of the velocity autocorrelation function (VACF, Supplementary Fig. 3B) of the ion adsorbed in the first layer [1]. τ is 433 fs for K^+ and 71 fs for Na^+. ΔG is 3.54 kcal/mol for K^+ and 8.89 kcal/mol for Na^+. The theoretical selectivity, defined the ratio of permeation rate of potassium ion over sodium ion, is 1295.

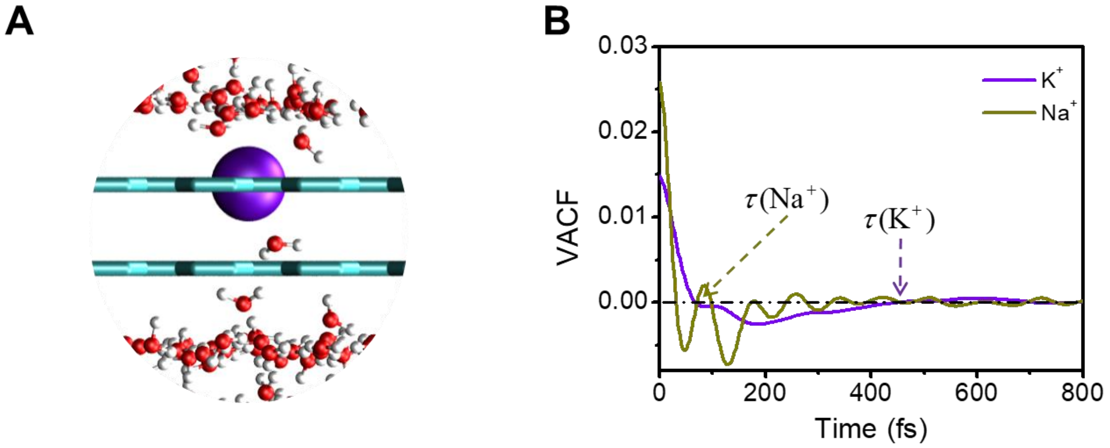

Supplementary Figure. 3. Determination of the vibration period (τ) of ion located at the first layer of nanopore. (A) Snapshot of the initial configuration, in which the ion was placed at the first layer of nanopore. (B) Velocity autocorrelation functions (VACFs) of K^+ and Na^+. The arrow indicates the location of corresponding τ.

Supplementary Text 4. Possible bi-layer nanopore configurations.

Supplementary Table 1. 25 bi-layer nanopore configurations from five types of single-layer nanopore. 13 bi-layer nanopores remain after excluding the duplications. Bilayer nanopores in gray color are not considered as they duplicate with the bi-layer nanopore inside corresponding parentheses.

	A0	A1	A2	A3	A4
A0	A0A0	A0A1	A0A2	A0A3	A0A4
A1	A1A0	A1A1	A1A2	A1A3	A1A4
A2	A2A0 (A1A3)	A2A1 (A1A2)	A2A2 (A1A1)	A2A3 (A1A0)	A2A4 (A1A4)
A3	A3A0 (A0A3)	A3A1 (A0A2)	A3A2 (A0A1)	A3A3 (A0A0)	A3A4 (A0A4)
A4	A4A0	A4A1	A4A2 (A4A1)	A4A3 (A4A0)	A4A4

Supplementary Text 5. Ion permeation through single-layer nanopore.

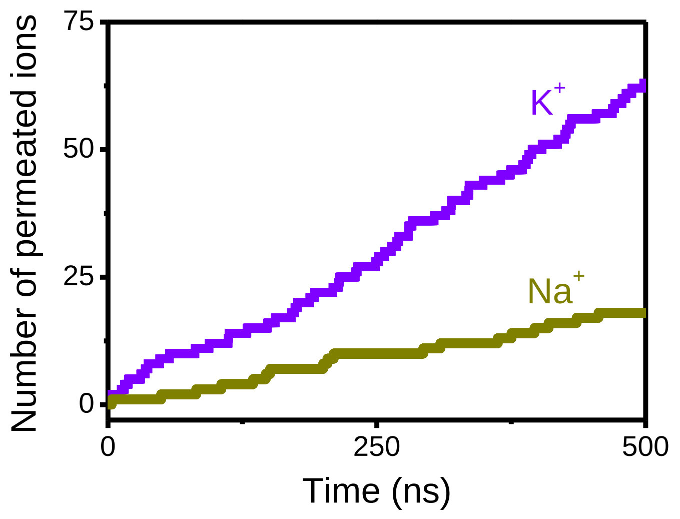

Supplementary Figure 4. Ion permeation test of single-layer nanopore, A0. Although K^+ shows a higher permeation rate than bi-layer nanopore, the leakage of Na^+ is severe and appears from the beginning, giving a selectivity ratio of only ~3.5, which agrees with previous simulation studies on single-layer nanopore [2-5].

Supplementary Text 6. Influence of flexible carbonyl groups on pore edge.

Here, we relaxed the flexibility of the carbonyl groups on the pore edge by removing freezing conditions. As shown in Supplementary Figure 5, the effective pore size can be well controlled. The change of the diagonal carbonyl-carbonyl distance is merely 0.66 Å and 0.20 Å in P1, and 0.01 Å and 0.21 Å in P2. In this case, strict K^+/Na^+ selectivity and the dual-ion transport mechanism can be well retained (Supplementary Figure 6).

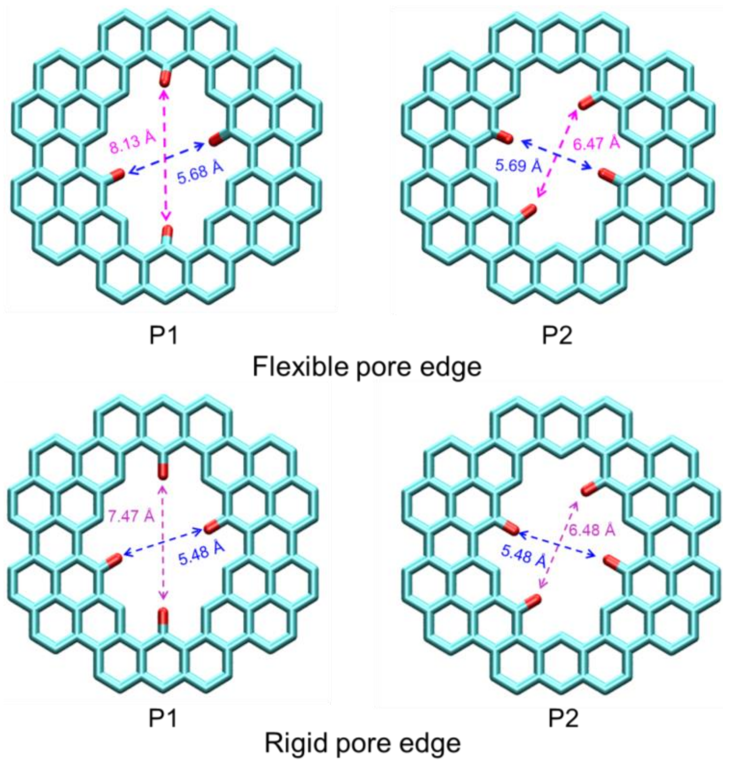

Supplementary Figure 5. Structures of the bi-layer graphene nanopores with flexible and rigid pore edge.

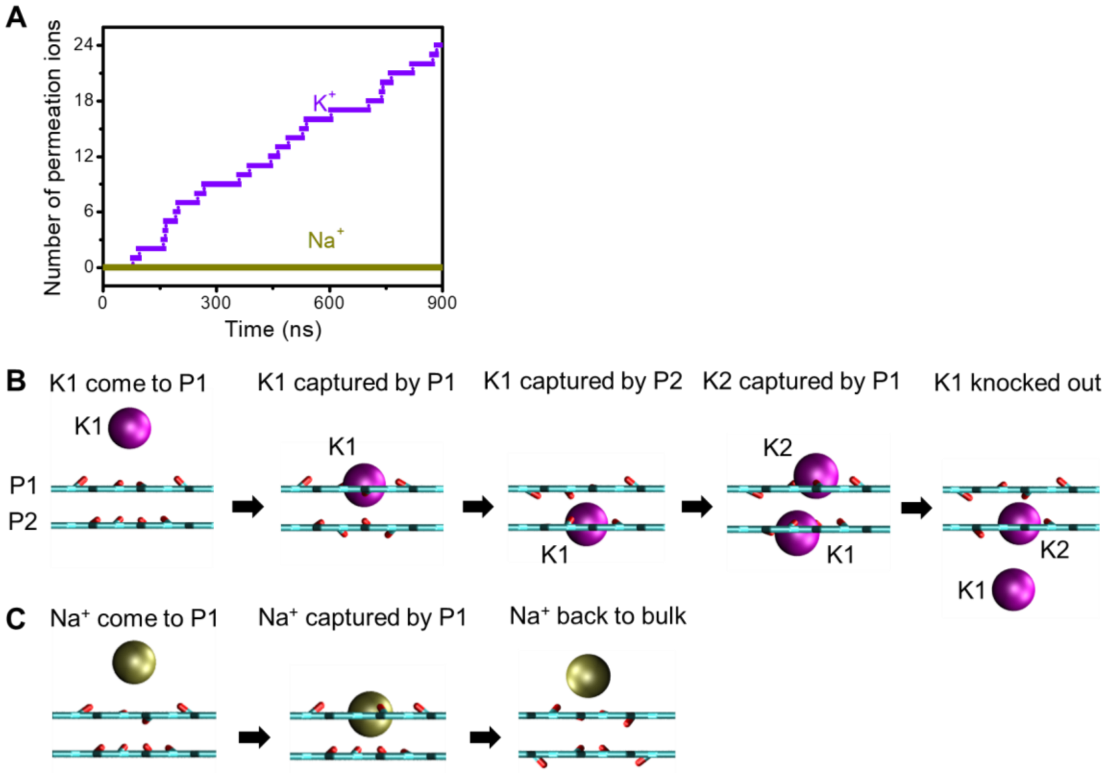

Supplementary Figure 6. Results on bi-layer graphene nanopores with flexible pore edge. (A) Ion permeation tests suggest strict K^+ selectivity. (B) Snapshots of the representative stages for K^+ permeation. The dual-ion transport mechanism is also discovered. (C) Snapshots of the representative stages for the blockade of Na^+. Other simulation parameters were identical to that used in the main text.

Supplementary Text 7. Coordination number of K^+ and Na^+.

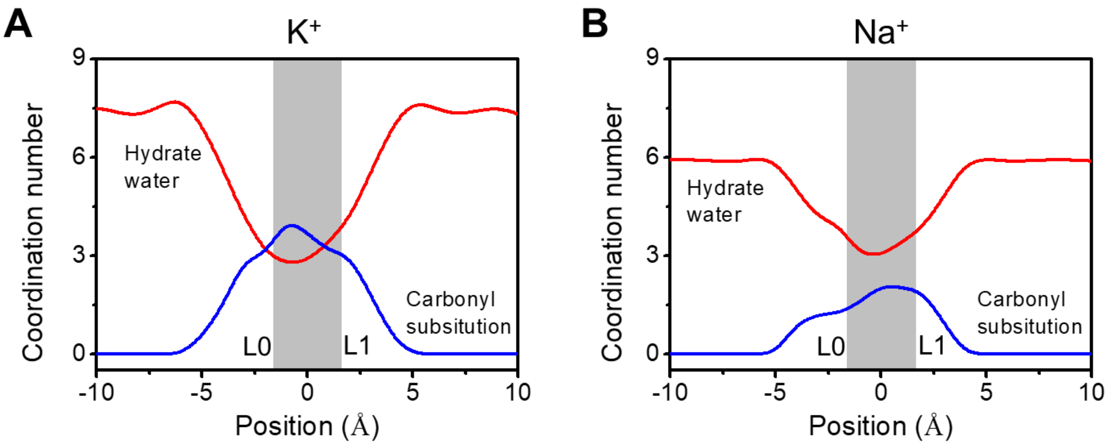

Supplementary Figure 7. Coordination number of (A) potassium and (B) sodium ion along nanopore, A0A1. A0A1 provides 4 carbonyls to substitute water in the solvation shell of K^+, whereas it provides only 2 carbonyls to Na^+, which underpins the higher energy barrier for Na^+ permeation than for K^+ permeation.

Supplementary Text 8. Electrostatic interaction energy of K-H_2O-K and K-2H_2O-K.

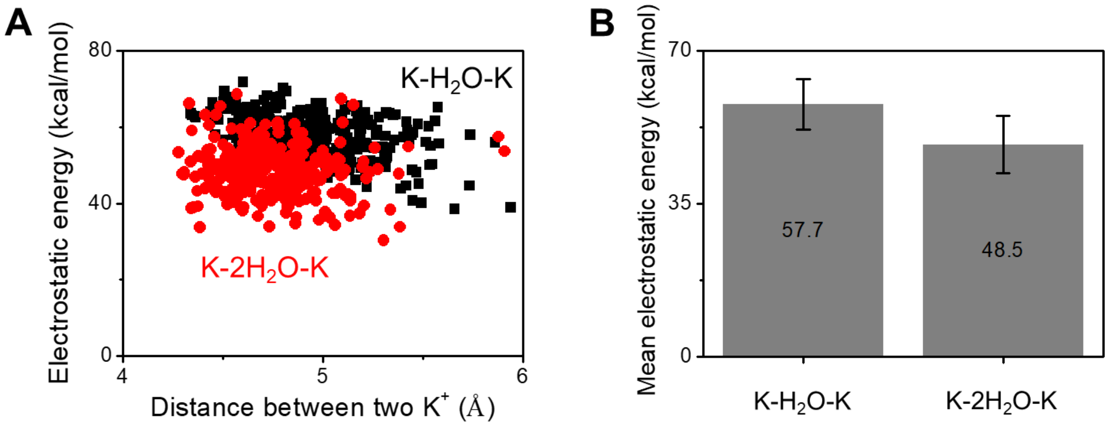

Supplementary Figure 8. Electrostatic interaction energy of K-H_2O-K and K-2H_2O-K. (A) Scattering plot of electrostatic interaction energy with the distance between two K^+. The calculation was achieved with CaFE [6]. (B) Mean electrostatic energy of K-H_2O-K and K-2H_2O-K.

Supplementary Text 9. Energy barrier of bi-layer nanopores.

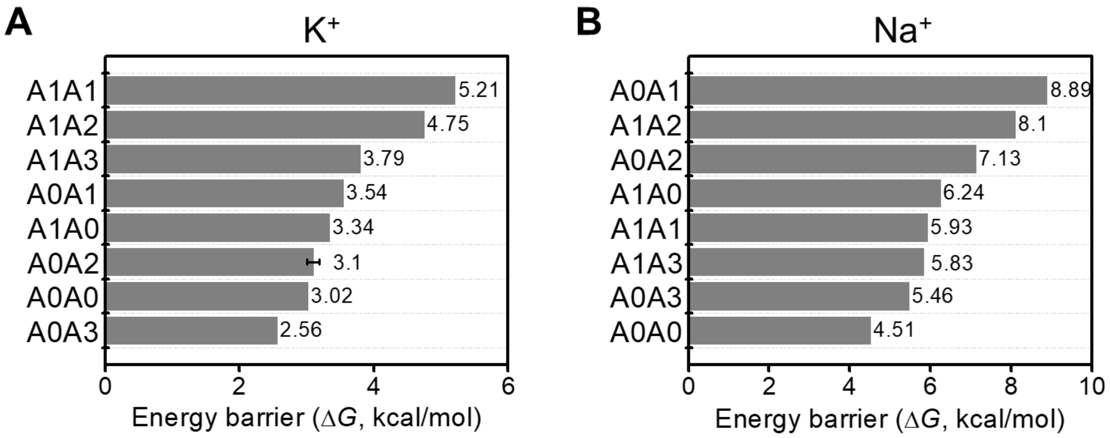

Supplementary Figure 9. Energy barrier of (A) K^+ and (B) Na^+ permeating through of bi-layer nanopores, A*m*A*n* (*m*=0 or 1; *n*=0-3). Energy barriers were obtained from PMF profiles (Fig. 2b in main text for A0A1, and not shown for other nanopores as they show a similar trend).

Supplementary Text 10. Ionic power generation.

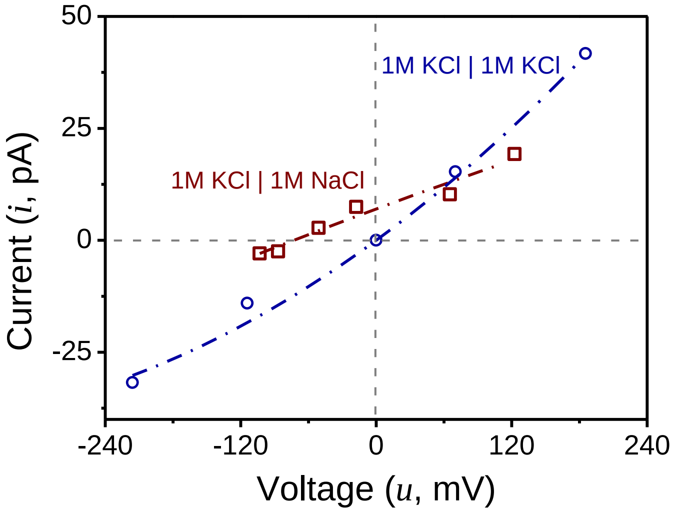

Supplementary Figure 10. Current-voltage curves from MD simulations for ionic power generation with bi-layer nanopores.

Supplementary Text 11. Estimation of reversal potential by a modified Goldman-Hodgkin-Katz theory.

Based on the Goldman–Hodgkin–Katz equation that describes ion current across a selective membrane [7], the current of substance A under uniform electric field is given by,

$$J_{\mathrm{A}} = Z_{\mathrm{A}}\mu \frac{D_{\mathrm{A}}}{L} \frac{[\mathrm{A}]_{\mathrm{R}} - [\mathrm{A}]_{\mathrm{L}}\exp(Z_{\mathrm{A}}\mu)}{1 - \exp(Z_{\mathrm{A}}\mu)}$$

where [A] is the concentration of substance A, D_{A} is the diffusion coefficient and treated as constant, Z_{A} is the charge valence, L is the thickness of membrane, and $\mu=FE_{\mathrm{m}}/RT$, E_{m} is the transmembrane potential difference, F is the Faraday's constant, R is the ideal gas constant, T is the temperature. The subscripts L and R donate left chamber and right chamber separated by the membrane.

For the system studied in this paper, when the total current of permeating ion is 0 (open circuit), we have,

$$J_{\mathrm{tot}} = J_{\mathrm{K^+}} + J_{\mathrm{Na^+}} + J_{\mathrm{Cl^-}} = 0$$

i.e.,

$$\exp(\frac{FE_{\mathrm{m}}}{RT}) = \frac{D_{\mathrm{K^+}}[\mathrm{K^+}]_{\mathrm{R}} + D_{\mathrm{Na^+}}[\mathrm{Na^+}]_{\mathrm{R}} + D_{\mathrm{Cl^-}}[\mathrm{Cl^-}]_{\mathrm{L}}}{D_{\mathrm{K^+}}[\mathrm{K^+}]_{\mathrm{L}} + D_{\mathrm{Na^+}}[\mathrm{Na^+}]_{\mathrm{L}} + D_{\mathrm{Cl^-}}[\mathrm{Cl^-}]_{\mathrm{R}}}$$

For a cation-selective membrane with $D_{\mathrm{Cl^-}}$=0, we can approximate $D_{\mathrm{K^+}}/D_{\mathrm{Na}}$ with the selectivity ratio (S), which represents the extent of sodium ion leakage. Then we obtain,

$$E_{\mathrm{m}} = \frac{RT}{F}\ln\left(\frac{S[\mathrm{K^+}]_{\mathrm{R}} + [\mathrm{Na^+}]_{\mathrm{R}}}{S[\mathrm{K^+}]_{\mathrm{L}} + [\mathrm{Na^+}]_{\mathrm{L}}}\right)$$

For the system studied in this paper, with pure KCl solution on the left and pure NaCl solution on the right initially, i.e. $[\mathrm{Na^+}]_{\mathrm{L}}=[\mathrm{K^+}]_{\mathrm{R}}=0$, we have,

$$E_{\mathrm{m}} = \frac{RT}{F}\ln\left(\frac{[\mathrm{Na^+}]_{\mathrm{R}}}{S[\mathrm{K^+}]_{\mathrm{L}}}\right)$$

Assuming the initial concentrations of the two solutions are the same and the activity coefficients of the two ions are the same, the equation can be further simplified to,

$$E_{\mathrm{m}} = \frac{RT}{F}\ln\left(\frac{1}{S}\right)$$

For the ionic power generation simulations, we reduced the interlayer distance to 3 Å to enhance the sampling of K^+ permeation events. This adjustment unavoidably sacrifices the K^+/Na^+ selectivity (S ~ 40-200). In this case, the corresponding E_{m} ranges from 96 to 137 mV, which is generally in agreement with the simulation result.

Supplementary Text 12. Comparison of the efficiency.

Supplementary Table 2. Efficiency of common energy conversion methods.

Method	Energy conversion efficiency	Reference
Natural gas	31-60%	[8]
Coal and oil	23-43%	[9]
Geothermal energy	7-10%	[10]
Wind energy	<59.3%	Betz's law
Salinity gradient power generation	49.3% (ion exchange membrane, charge selectivity ~99.3%)	[11]
PoPee-OPG	37.5%	this work

Supplementary Text 13. Effect of confining cylinder radius on PMF.

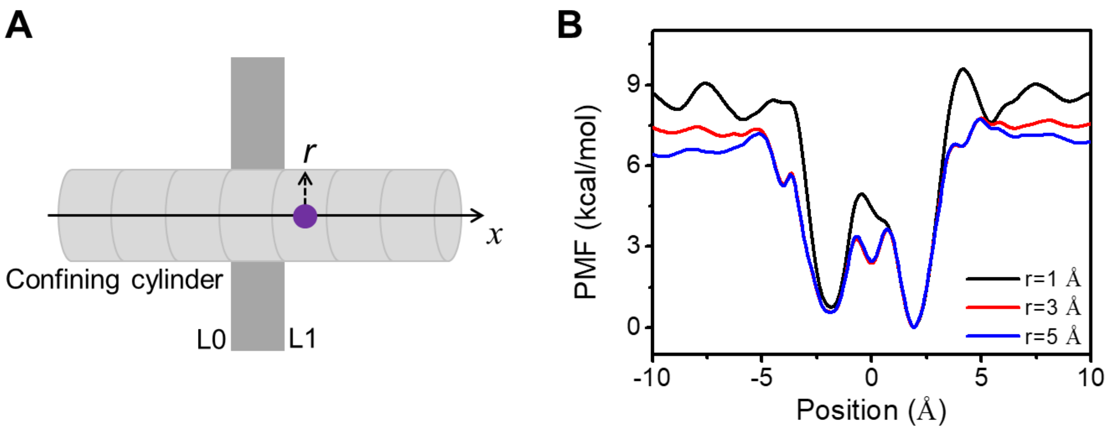

Supplementary Figure 11. The effect of confining cylinder radius on PMF sampling. (A) The scheme of pulling ion through nanopore. (B) The effect of radius of confining cylinder on PMF of K^+ through bi-layer nanopore, A0A2. Obviously the radius of 1 Å is too narrow and gives unreal PMF. The PMF profiles barely change when the radius changes from 3 Å to 5 Å. Therefore, we use a confining cylinder with radius of 5 Å when sampling the PMFs.

Supplementary Text 14. Simulation system for ionic power generation.

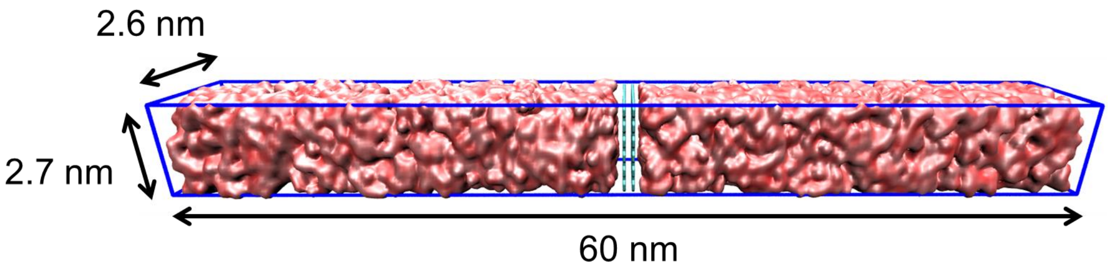

Supplementary Figure 12. Simulation set up for the ionic power generation. The dimensions of the simulation system are not to scale.

Supplementary Text 15. Free energy change of mixing solutions.

We model the salt solution with a primitive model. The solvent is treated implicitly, acting as a continuous medium with a dielectric constant of 80. The ions are modeled as charged hard spheres of different diameters. We consider a solution that contains KCl and NaCl, giving three types of ions: K^+, Na^+, and Cl^-. For an electrolyte solution of volume V, the free energy (F) is given by,

$$F = V\sum_i c_i\mu_i$$

where c_i and μ_i are the concentration and chemical potential of ion of type i. For the systems considered in this work, μ_i contains four terms,

$$\mu_i = \mu_i^0 + \mu_i^{\mathrm{id}} + \mu_i^{\mathrm{hs}} + \mu_i^{\mathrm{el}}$$

where μ_i^0 is a constant and does not affect the calculations in this work. Therefore, we set $\mu_i^0 = 0$ for all ion types. μ_i^{id} is the contribution due to the translational entropy and is given by $\mu_i^{\mathrm{id}} = \ln c_i - 1$ for the bulk solution, μ_i^{hs} accounts for the hard-sphere exclusion among ions and is estimated with the modified fundamental measure theory, and μ_i^{el} is due to the electrostatic correlations and is estimated by mean spherical approximation. More details about the hard sphere exclusion term and electrostatic correlation term are referred to our previous work [12, 13].

References:

1. Tully, J.C., G.H. Gilmer, and M. Shugard, *Molecular dynamics of surface diffusion. I. The motion of adatoms and clusters.* The Journal of Chemical Physics, 1979. **71**(4): p. 1630-1642.
2. He, Z., et al., *Bioinspired graphene nanopores with voltage-tunable ion selectivity for Na(+) and K(+).* ACS Nano, 2013. **7**(11): p. 10148-10157.
3. Fang, A., et al., *Highly mechanosensitive ion channels from graphene-embedded crown ethers.* Nature Materials, 2019. **18**(1): p. 76-81.
4. Chen, Y.J., et al., *Molecular insights into multilayer 18-crown-6-like graphene nanopores for K+/Na+ separation: A molecular dynamics study.* Carbon, 2019. **144**: p. 32-42.
5. Gong, K., et al., *Voltage-gated multilayer graphene nanochannel for K+/Na+ separation: A molecular dynamics study.* Journal of Molecular Liquids, 2020. **317**: p. 114025.
6. Liu, H. and T. Hou, *CaFE: a tool for binding affinity prediction using end-point free energy methods.* Bioinformatics, 2016. **32**(14): p. 2216-2218.
7. Junge, D., *Nerve and Muscle Excitation*. 1992: Oxford University Press, Incorporated.
8. Storm, K., *Chapter 6 - Combined cycle power plant (1×1) labor estimate*, in *Industrial Construction Estimating Manual*, K. Storm, Editor. 2020, Gulf Professional Publishing. p. 95-159.
9. Trudeau, N. and M. Francoeur, *Energy efficiency indicators for public electricity production from fossil fuels.* Oil Market Report Internation Energy Agency, 2008. **1**: p. 1-23.
10. Schavemaker, P. and L. Van der Sluis, *Electrical power system essentials*. 2017: John Wiley & Sons.
11. Gao, J., et al., *High-Performance Ionic Diode Membrane for Salinity Gradient Power Generation.* Journal of the American Chemical Society, 2014. **136**(35): p. 12265-12272.
12. Li, Z. and J. Wu, *Density-functional theory for the structures and thermodynamic properties of highly asymmetric electrolyte and neutral component mixtures.* Physical Review E, 2004. **70**(3): p. 031109-031101.
13. Kong, X., et al., *Molecular Theory for Electrokinetic Transport in pH-Regulated Nanochannels.* Journal of Physical Chemistry Letters, 2014. **5**(17): p. 3015-3020.
